# Efficacy and safety of roxadustat for the treatment of anemia in non-dialysis chronic kidney disease patients: A systematic review and meta-analysis of randomized double-blind controlled clinical trials

**DOI:** 10.3389/fnut.2022.1029432

**Published:** 2022-11-04

**Authors:** Ting Chen, Junyue Huang, Hui Dong, Lili Xu, Caihe Chen, Yu Tang, Wenhui Huang

**Affiliations:** ^1^The First Clinical Medical College, Gansu University of Traditional Chinese Medicine, Lanzhou, China; ^2^Department of Nephrology, Gansu Provincial Hospital, Lanzhou, China; ^3^Clinical Lab, Gansu Provincial Hospital, Lanzhou, China

**Keywords:** chronic kidney disease, non-dialysis, anemia, roxadustat, meta-analysis, efficacy, hemoglobin, clinical trials

## Abstract

**Objective:**

To evaluate the efficacy and safety of roxadustat in the treatment of anemia in non-dialysis-dependent chronic kidney disease (NDD-CKD) patients.

**Materials and methods:**

For this systematic review and meta-analysis, we searched for randomized controlled trials (RCTs) of anemia in NDD-CKD patients to assess the efficacy and safety of roxadustat. The primary efficacy endpoint was the proportion of patients who achieved a hemoglobin (Hb) response. Secondary efficacy endpoints were hepcidin, serum iron, serum ferritin (SF), total iron-binding capacity (TIBC), transferrin saturation (TAST), and low-density lipoprotein (LDL). In addition, adverse events (AEs) were compared. Meta-analyses were performed using Revman 5.4 software. The quality of the evidence was assessed using the Cochrane risk of bias tool. This study was conducted under a pre-established protocol registered with PROSPERO (registration number: CRD42021252331).

**Results:**

Seven studies enrolled 4,764 patients, of whom 2,730 received roxadustat and 2,034 received placebo. The results of this meta-analysis showed that roxadustat increased Hb levels [weighted mean difference (WMD) = 1.43, 95% CI: 1.17 to 1.68, *P* < 0.001, *I*^2^ = 95%], and Hb response [relative ratio (RR) = 8.12, 95% CI: 5.80 to 11.37, *P* < 0.001, *I*^2^ = 61%]. In addition, roxadustat significantly increased transferrin TAST. During the treatment period in patients with anemia, the AEs of roxadustat compared with placebo was not statistically significant.

**Conclusion:**

Roxadustat can improve anemia in NDD-CKD patients by increasing Hb levels and regulating iron metabolism, but does not increase the incidence of AEs.

**Systematic review registration:**

[https://www.crd.york.ac.uk/prospero/], identifier [CRD42021252331].

## Introduction

Anemia is one of the most common complications observed in patients with chronic kidney disease (CKD) (1), and leads to increased cardiovascular morbidity and mortality. At the same time, patients’ quality life is significantly reduced (2, 3).

Erythropoiesis stimulating agents (ESAs) and iron are recommended to improve anemia in CKD patients (4). Studies have shown that by maintaining hemoglobin (Hb) at 100–110 g/L, ESA can significantly reduce the need for blood transfusions. However, ESA increases the risk of cardiovascular and cerebrovascular events, thrombosis, end-stage renal disease (ESRD), and death if higher Hb levels (130 g/L) are targeted (5, 6). Iron use may also lead to allergic reactions or delay complications, such as serious infections or cardiovascular events that interfere with treatment (7–9), all of these have implications for the management of patients with CKD anemia.

Hypoxia-inducible factor (HIF) is a heterodimer composed of alpha and beta subunits that activates erythropoietin (EPO) gene transcription. The prolyl hydroxylase domain (PHD) is a key regulator of HIF, sensing oxygen levels and controlling HIF activity. When PHD activity is reduced under hypoxia, the reduced hydroxylation of HIF-α stabilizes it and enters the nucleus, where it dimerizes with HIF-β, binds to hypoxia-responsive elements, induces gene expression, and finally stimulates internal Production of source EPO. production, improve iron metabolism, and promote erythropoiesis (10). Hypoxia-inducible factor Prolyl hydroxylase inhibitors (HIF-PHIs) are a new class of drugs for the treatment of anemia that mimic hypoxic environments, one of which is roxadustat. They differ from ESAs in that they do not directly activate the EPO receptor, but stimulate the production of endogenous EPO in the kidney and the liver. Also, they are administered orally rather than parenterally (11). Based on the above properties, these drugs have good prospects in the treatment of CKD-related anemia. Therefore, it is necessary to further study the efficacy and safety of roxadustat in the treatment of anemia. There are currently more than 15 randomized controlled trials (RCTs) and approximately 10,000 registered patients worldwide, including non-dialysis-dependent (NDD) and dialysis-dependent (DD) patients. Two meta-analyses (12, 13) of a total of 258 NDD-CKD patients examined the effectiveness of roxadustat and showed that roxadustat significantly improved Hb levels and iron metabolism compared with placebo. This meta-analysis will include additional studies to assess the efficacy and safety of roxadustat in patients with NDD-CKD. We present the following articles/cases based on the PRISMA report checklist.

## Materials and methods

### Data sources and searches

We searched the following major research databases: PubMed, Web of Science, Embase, and The Cochrane Library. Search for renal disease using the keywords: “chronic kidney disease,” “chronic renal disease,” “renal insufficiency, chronic,” “chronic kidney insufficiencies,” “anemia of renal failure,” and “renal anemia.” Search for roxadustat using the following keywords: “FG-4592,” “roxadustat,” “hypoxia-inducible factor prolyl hydroxylase inhibitor,” and “HIF-PHI.”

### Study selection

The following inclusion criteria were used for full-text screening: (1) the population consisted of NDD-CKD anemia patients with unlimited CKD stages; (2) the intervention group received roxadustat regardless of dose and duration; (3) the control group was placebo; and (4) The type of study was a RCT. The exclusion criteria are as follows: (1) The focus of the study is anemia secondary to other causes, such as malignant tumors, blood diseases or aplastic anemia; (2) CKD patients requiring dialysis; (3) Comments, opinions and case reports. (4) Repeated publications; and (5) Patients receiving ESA treatment.

### Outcome measures

The outcomes of this systematic review were changes in Hb response rate, Hb parameters, hepcidin, serum iron, serum ferritin (SF), total iron-binding capacity (TIBC), transferrin saturation (TAST), and low-density lipoprotein (LDL) cholesterol. In this study, Hb response was defined as Hb ≥ 11.0 g/dl that increased from baseline by ≥1.0 g/dl in patients with Hb > 8.0 g/dl or ≥2.0 g/dl in patients with baseline Hb ≤ 8.0 g/dl, at any given time.

### Data extraction and assessment of risk of bias

Two authors independently extracted data and resolved differences in consultation with the other author. Standardized tables were used to clear the following data: patient demographics, study design, drug intake and dose, duration of follow-up, outcome indices (Hb response, LDL cholesterol, hepcidin, etc.) and adverse events (AEs) (hypertension, hyperkalemia, symptoms digestive system and respiratory system, etc.).

Two authors independently assessed the risk of bias at the outcome level for each included study using the risk of bias assessment tool developed by the Cochrane Bias Methods Group. The evaluation included the following items: (1) random sequence generation, (2) allocation concealment, (3) blinding of patients, (4) blinding of outcome assessment, (5) completeness of outcome data, (6) selective reporting, and (7) other biases.

### Statistical analyses

The weighted mean difference (WMD) were calculated for the effects of roxadustat on outcomes. AEs use relative ratios (RRs); each effect size is expressed with a 95% CI. The *Q*-test and *I*^2^ values were used to assess heterogeneity across studies. If *I*^2^-value < 50%, *P*-value > 0.05, the heterogeneity is considered low, using the fixed-effect model. Otherwise, the heterogeneity is considered high, using the random-effect model. Given the differences in the length of the study, we performed a subgroup analysis of the hemoglobin parameters. The funnel plot was conducted to evaluate the likelihood of publication bias. Sensitivity analyses were conducted by removing one study at a time. All statistical analyses were performed using Revman 5.4; statistical significance is defined as *P* < 0.05.

## Results

### Search results

**FIGURE 1:**
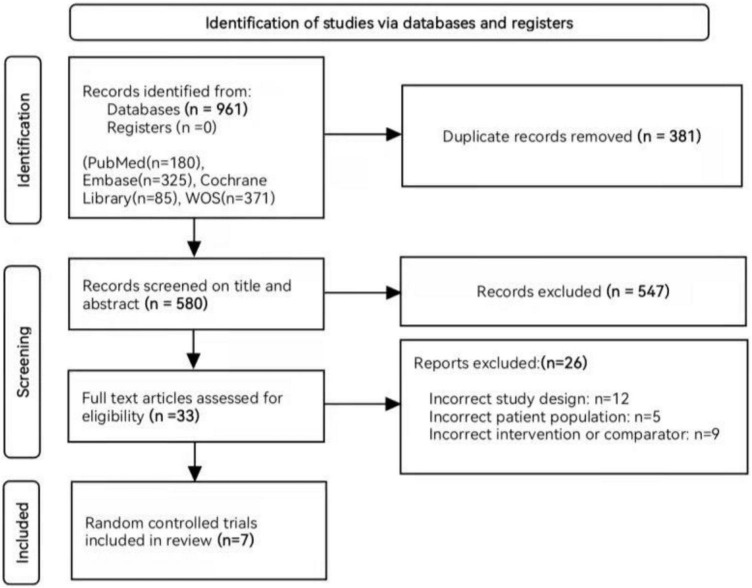
Flow chart of literature search and selection.

The preliminary search strategy identified 961 unique records, of which 33 were potentially eligible after filtering the title and abstract. Finally, this meta-analysis included seven RCTs involving a total of 4,764 patients with NDD-CKD (14–20). A flowchart for study selection and reasons for exclusion is shown in Figure 1.

### Study characteristics

All included studies were RCTs, including four phase III clinical studies (14–17), and three phase II clinical studies (18–20) (Table 1). In a study of NDD-CKD and DD-CKD patients, we extracted NDD-CKD-related indicators (19). Before treatment, all patients did not receive ESA; five studies did not allow intravenous (IV) or oral iron, one study allowed IV or oral iron, and one study did not mention iron supplementation; during treatment and follow-up, all patients were allowed IV or oral iron. The total number of patients was 4,764 NDD-CKD patients. the control group received a placebo. Five studies examined patients with CKD stages 3–5, and one study examined patients with CKD stages 2–5. Follow-up periods ranged from 6 to 104 weeks. The primary efficacy endpoints were the mean change in Hb from baseline to the end of the treatment period and the proportion of patients who achieved a Hb response.

**TABLE 1:** Characteristics of the included studies in the meta-analysis.

Study (year)	Female n (%)	Age mean age, y		Sample size		Hb baseline		Site	Study type	CKD stage	Study drug administration	Duration (weeks)	Clinical trials
								
	
T	C	T	C	T	C						
Shutov et al. (14)	326 (55.16)	62.0 (20–89)	63.0 (26–90)	391	201	9.08 (0.76)	9.10 (0.72)	Europe	Multicenter, randomized, double-blind, placebo-controlled	3 to 5	Weight ł45 to ≥70 kg = 70 mg; weight >70 to ≥160 k*g* = 100 mg, oral, TIW	52–104	III
Fishbane et al. (17)	1594 (57.31)	60.9 (14.7)	62.4 (14.1)	1393	1388	9.1 (0.7)	9.1 (0.7)	Europe	Multicenter, randomized, double-blind,	3 to 5	40–100 mg, oral, TIW	52–104	III
									placebo-controlled				
Coyne et al. (15)	551 (59.76)	64.9 (12.6)	64.8 (13.2)	616	306	9.1 (0.75)	9.09 (0.69)	Europe	Randomized, double-blind, placebo-controlled	3 to 5	Weight ł45 to <70 kg = 70 mg.; weighing >70 kg = 100 mg, oral, TIW	28–52	III
Chen et al. (16)	95 (62.91)	54.7 (13.3)	53.2 (13.1)	101	50	8.9 (0.8)	8.9 (0.7)	Asia	Initial 8-week, double-blind, placebo-controlled phase. After	3 to 5	Weight ł40 to <60 kg = 70 mg; weight ł60 kg = 100 mg, oral, TIW	8–26	III
									received roxadustat during an 18-week				
									Open-label phase.				
Akizawa et al. (18)	57 (53.27)	65.0 (8.5)	61.9 (10.6)	80	27	9.4 (0.6)	9.3 (0.7)	Asia	Randomized, parallel-group, double-blind	2 to 5	50, 70, or 100 mg, oral, TIW	18–24	II
									placebo-controlled				
Chen et al. (19)	54 (59.34)	49.6 (14.8)	51.4 (11.9)	61	30	8.8 (0.9)	8.9 (0.8)	Asia	Multicentric, randomized, double-blind, placebo-controlled	3 to 5	weight 40–60 kg, >60 to 80 mg or >80 to 100 mg, oral, TIW	6	II
Besarab et al. (20)	326 (55.16)	64	68.6	88	28	10.3 (0.9)	10.3 (0.9)	Europe	Multicenter, single-blind, randomized placebo-controlled	3 to 4	1.0, 1.5, 2.0, and then 0.7 mg/kg, oral, BIW or TIW	20	II

T, experimental; C, control; TIW, three times weekly; BIW, twice times weekly.

### Assessment of the risk of bias

**FIGURE 2:**
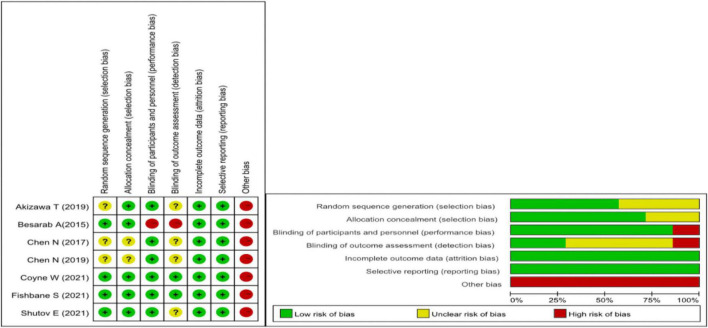
Cochrane risk of bias assessment.

We used the Cochrane Collaboration tool to assess the risk of bias. Due to all the research funded by medical companies, we believe there is a high risk of bias. Figure 2 for the specific assessment of the risk of bias.

### Primary outcomes

#### Hemoglobin

**FIGURE 3:**
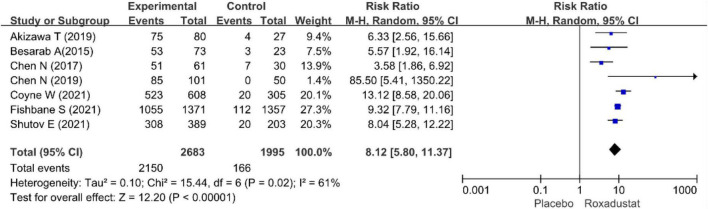
Hemoglobin response.

**FIGURE 4:**
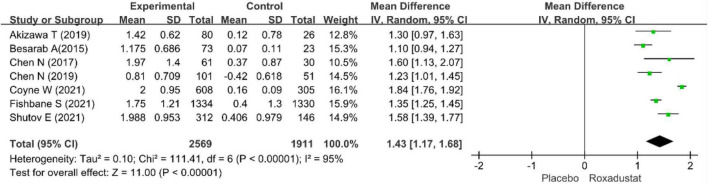
Mean change in hemoglobin.

The primary efficacy endpoints were the mean change in Hb levels from baseline to the end of the treatment period and the proportion of patients who achieved a Hb response. Hb responses were reported in all included studies (*n* = 4,678 patients) (14–20) and showed significantly higher Hb responses to roxadustat than to placebo (RR = 8.12, 95% CI: 5.80 to 11.37, *P* < 0.001; *I*^2^ = 61%, *P* = 0.02) (Figure 3). All included studies (*n* = 4,480 patients) (14–20) assessed changes from baseline in Hb levels in patients treated with roxadustat and placebo (WMD = 1.43, 95% CI: 1.17 to 1.68, *P* < 0.001; *I*^2^ = 95%, *P* < 0.001) (Figure 4).

**FIGURE 5:**
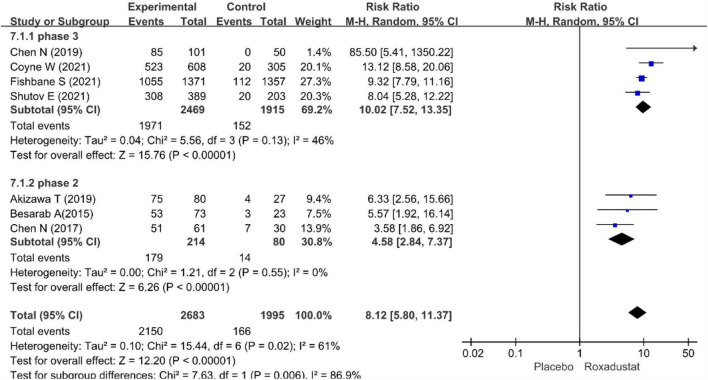
Sub-grouped of hemoglobin response.

**FIGURE 6:**
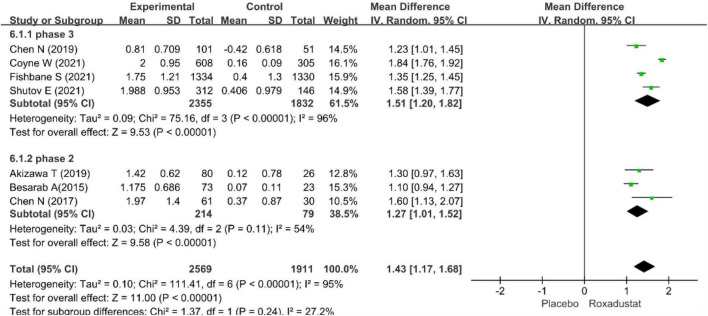
Sub-grouped of mean change in hemoglobin.

The pool results showed that roxadustat was effective in improving Hb regardless of the length of the study. In a subset of NDD-CKD patients in a phase III clinical trial (14–17), Hb levels were significantly increased with roxadustat compared with placebo (WMD = 1.51, 95% CI: 1.20–1.82, *P* < 0.001; *I*^2^ = 96%, *P* < 0.001) the results were similar in the ? phase of the study (18–20) (WMD = 1.43, 95% CI: 1.17–1.68, *P* < 0.001; *I*^2^ = 54%, *P* = 0.11) (Figures 5, 6).

### Secondary outcomes

#### Low-density lipoprotein-cholesterol

**FIGURE 7:**
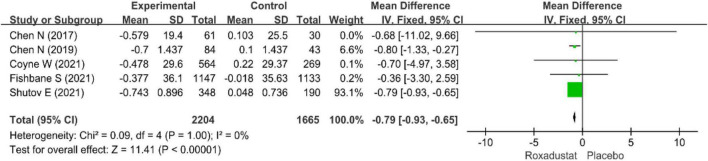
Low-density lipoprotein (LDL).

Five studies (14–17, 19) (*n* = 3,869 patients) examined changes in LDL-cholesterol in patients with CKD anemia (*n* = 3,869 patients), and after treatment with roxadustat, the change in LDL-cholesterol was significantly lower than in the placebo group (WMD = −0.79, 95% CI: −0.93 to −0.65, *P* < 0.001; *I*^2^ = 0%, *P* = 1.00) (Figure 7).

#### Hepcidin

**FIGURE 8:**
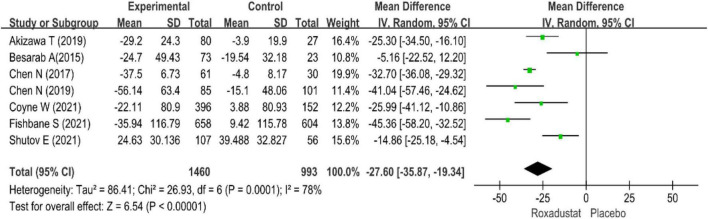
Hepcidin.

All studies evaluated hepcidin in NDD-CKD patients treated with roxadustat (14–20), hepcidin was significantly lower than placebo when treated with roxadustat (WMD = −27.60, 95% CI: −35.87 to −19.34, *P* < 0.001; *I*^2^ = 78%, *P* < 0.001) (Figure 8).

#### Serum iron

**FIGURE 9:**
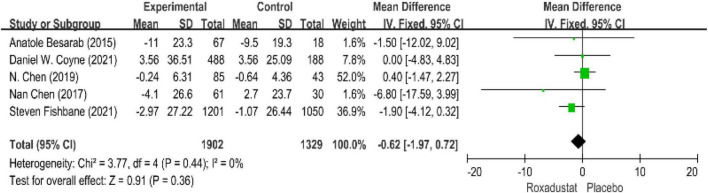
Serum iron.

Five studies evaluated serum iron levels in patients with anemia receiving roxadustat (*n* = 3,294 patients) (15–17, 19, 20) and serum iron levels in the roxadustat group were not significantly different from those in the placebo group, the difference (WMD = −0.62, 95% CI: −1.97 to 0.72, *P* = 0.36; *I*^2^ = 0%, *P* = 0.44) (Figure 9).

#### Serum ferritin

**FIGURE 10:**
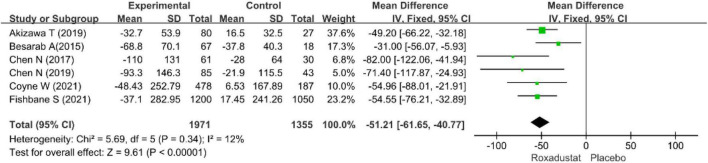
Serum ferritin (SF).

Six studies (15–20) assessed SF (*n* = 3326) in roxadustat treated patients with CKD anemia, and SF was significantly lower than placebo after roxadustat treatment (WMD = −51.21, 95% CI: −61.65 to −45.77, *P* = < 0.001; *I*^2^ = 12%, *P* = 0.34) (Figure 10).

#### Total iron-binding capacity

**FIGURE 11:**
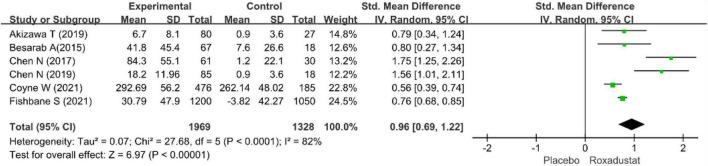
Total iron-binding capacity (TIBC).

Six studies evaluated TIBC in CKD anemia patients treated with roxadustat (15–20) (*n* = 3,351 patients) and TIBC was significantly higher in the roxadustat-treated group than in the placebo group (WMD = 0.96, 95% CI: 0.69 to 1.22, *P* < 0.001; *I*^2^ = 68%, *P* = 0.07) (Figure 11).

#### TSAT

**FIGURE 12:**
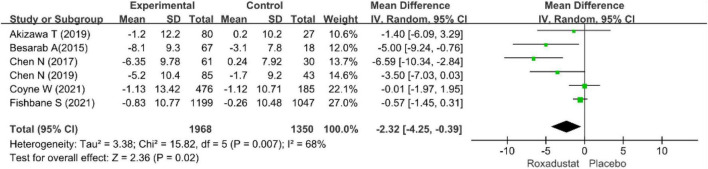
Trasferrin saturation (TSAT).

Six studies examined TSAT in patients with CKD anemia receiving roxadustat (15–20), and TSAT was significantly lower in the roxadustat treated group than in the placebo group (WMD = −2.32, 95% CI: −4.25 to −0.39, *P* = 0.02; *I*^2^ = 68%, *P* = 0.007) (Figure 12).

#### Adverse effects

**FIGURE 13:**
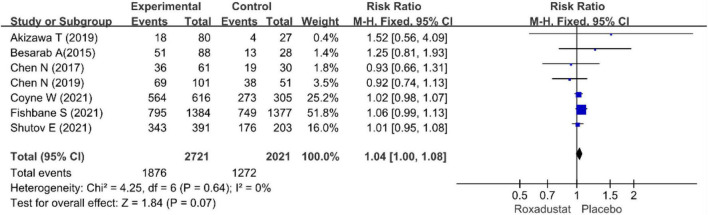
Treatment-emergent adverse event (TEAE).

**FIGURE 14:**
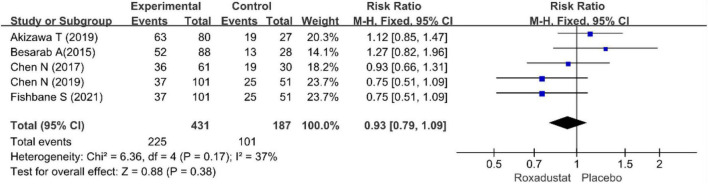
Adverse events (AEs).

We performed separate meta-analyses for ESRD, hypertension, nausea, vomiting, diarrhea, constipation, hyperkalemia, peripheral edema, dizziness, and nausea. ESRD, dizziness, headache, cough, viral upper respiratory tract infection, upper respiratory tract infection, pruritus, urinary tract infection, and vomiting were not statistically significant. Differences between treatment groups included hypertension, nausea, and diarrhea. Table 2 lists the AEs. Overall, roxadustat was not statistically significant in safety compared with placebo treatment-emergent adverse events (TEAEs: RR = 1.04, 95% CI: 1.00−1.08, *P* = 0.64; *I*^2^ = 0%, *P* = 0.84 and AEs: RR = 0.93, 95% CI: 0.79−1.09, *P* = 0.16; *I*^2^ = 37%, *P* = 0.17) (Figures 13, 14).

**TABLE 2:** Adverse events analysis.

Indicators	Number of studies	*Q*-test *P*-value	*I*^2^ (%)	Model selected	RR (95% CI)	*P*-value
ESRD	4	0.16	46	Fixed	1.10 (0.97, 1.24)	0.13
Hypertension	5	0.54	0	Fixed	1.43 (1.20, 1.70)	< *0*.001
Edema peripheral	5	0.85	0	Fixed	1.35 (1.13, 1.63)	0.001
Dizziness	5	0.33	13	Fixed	0.84 (0.66, 1.06)	0.13
Hyperkalaemia	6	0.94	0	Fixed	1.32 (1.09, 1.59	0.004
Nausea	5	0.22	31	Fixed	1.39 (1.13, 1.70)	0.002
Diarrhea	3	0.78	0	Fixed	1.57 (0.54, 4.53)	0.4
Headache	5	0.93	0	Fixed	1.17 (0.94, 1.47)	0.16
Cough	3	0.08	61	Random	1.16 (0.69, 1.93)	0.58
Viral upper respiratory tract infection	3	0.07	62	Random	1.22 (0.84, 1.76)	0.29
Upper respiratory tract infection	4	0.17	40	Fixed	1.03 (0.83, 1.27)	0.8
Pruritus	3	0.02	75	Random	1.37 (0.69, 2.73)	0.36
Asthenia	3	0.52	0	Fixed	1.02 (0.78, 1.32)	0.9
Urinary tract infection	3	0.03	72	Random	1.20 (0.69, 2.09)	0.51
Vomiting	2	0.38	0	Fixed	1.13 (0.83, 1.54)	0.44

#### Publication bias

**FIGURE 15:**
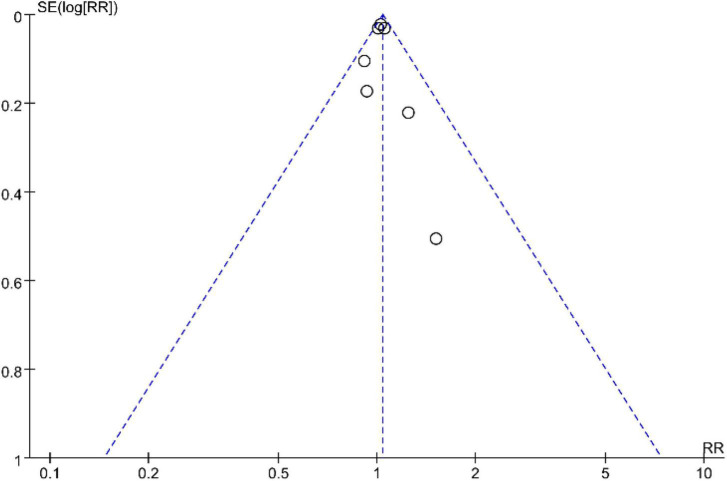
Funnel plot.

In this study, a funnel plot was drawn for tea and the results showed a symmetric distribution, suggesting less potential for publication bias (Figure 15).

## Discussion

**FIGURE 16:**
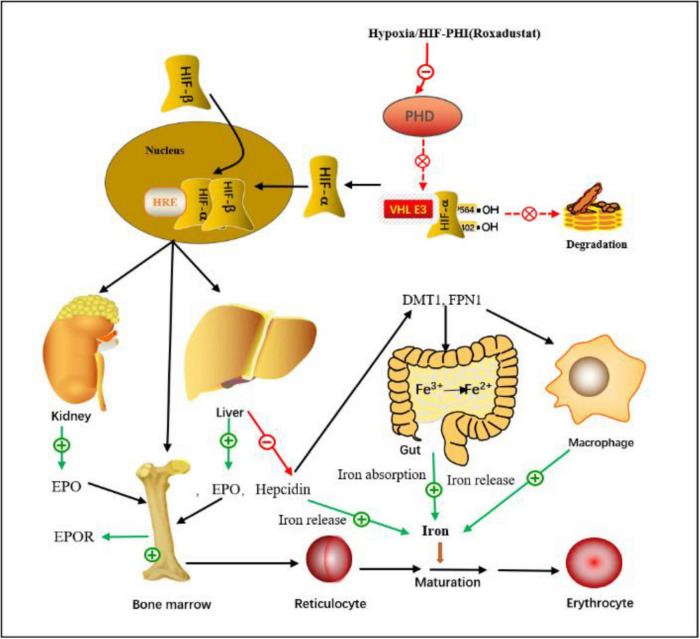
The mechanism of action of the HIF-PHI **(Roxadustat)**. Roxadustat inhibits the PHD-induced HIF degradation by simulating the hypoxic environment *in vivo*, so that HIF-α accumulates in cells, then enters the nucleus, binds to HIF-β, and forms a dimer complex, after a series of biological activities, it acts on various target organs such as liver, kidney and bone marrow, increased EPO expression and EPOR activation, and regulated iron absorption and iron release, ultimately increased erythrocyte synthesis. HIF, hypoxia-inducible factor; HRE, hypoxia response element; PHD, propyl-4-hydroxylase domain protein; VHL, von Hippel-Lindau protein; DMT1, divalent metal transporter-1; FPN1, ferroportin-1; EPO, erythropoietin; EPOR, erythropoietin receptor.

Anemia in CKD is a common problem for several reasons (21), mainly due to insufficient EPO production and iron deficiency; other causes include chronic blood loss, inflammation, uremic toxins, and malnutrition. Approximately 90% of EPO is produced by renal erythropoietin-producing cells (REPCs) in the kidneys, and EPO production is regulated by HIF (22, 23). The kidneys are prone to hypoxemia (24). Hypoxemia induces EPO expression to control HIF-mediated erythrocyte and oxygen content (22). HIF is an essential cellular response factor to hypoxia, consisting of α and β subunits, and plays a central role in erythropoiesis (25–27). HIF activates EPO gene transcription and acts on some genes that affect iron absorption and transcription, thereby synergistically promotes erythropoiesis, including the production of endogenous EPO, regulates iron absorption and storage, increases iron bioavailability and promotes erythrocyte maturation. PHD is the rate-limiting enzyme of HIF degradation, and the activity of HIF is regulated by PHD, PHD-inhibitor can activate the HIF pathway and promote the production of EPO, thereby increasing the level of Hb (22, 23, 27) (Figure 16).

This meta-analysis evaluated the efficacy and safety of roxadustat for the treatment of anemia in patients with NDD-CKD. The primary efficacy endpoint was the change in Hb and Hb response, where Hb response defined as Hb ≥ 11.0 g/dL that increased from baseline by ≥1.0 g/dL in patients with Hb > 8.0 g/dL or ≥2.0 g/dL in patients with baseline Hb ≤ 8.0 g/dL at any time point; study results showed that compared with placebo, the Hb response was significantly higher in the Roxadustat group. At the same time, after roxadustat treatment, compared with the placebo group, the Hb level after roxadustat treatment was significantly higher than the baseline value, and the iron metabolism-related indicators were significantly improved. Therefore, roxadustat can improve the Hb levels and iron metabolism, thereby correcting CKD-related anemia.

Iron is the main element for the synthesis of Hb, and the human body needs 20–25 mg of iron per day to maintain the synthesis of hemoglobin. Iron deficiency anemia (IDA) is common in patients with CKD and is associated with an increased risk of morbidity and mortality (28). The metabolism of iron requires the participation of various enzymes, including hepcidin secreted by hepatocytes, which can inhibit iron absorption and release iron stored in the body (29). The level of hepcidin in CKD patients is higher than that in the normal population, and roxadustat can reduce the level of hepcidin in patients and increase the absorption and utilization of iron. In addition, studies have shown that additional iron supplementation is not required when using this drug (30). However, our results showed a decrease in TSAT and SF, while an increase in TIBC, indicating insufficient iron stores and available iron in the body. Included RCTs may have had lower iron supplementation during treatment. Therefore, adequate iron supplementation with roxadustat can achieve better therapeutic effects. However, the included RCTs did not have data on iron supplementation, so we did not perform an analysis of iron supplementation. There is currently no standard iron therapy in clinical practice. The Asia Pacific Society of Nephrology (APSN) recommends a ferritin value > 100 ng/ml and a TSAT > 20% (31).

In addition, roxadustat has other potential therapeutic effects, including modulation of lipoprotein metabolism, delaying the progression of CKD (32), and the potential to treat ESA hyporesponsive anemia. Of concern is the treatment of ESAs hyporesponsiveness. Inflammation and hepcidin cause abnormal iron mobilization and absorption, which are the main causes of refractory anemia in ESRD (5, 33). A Previous study found a significant increase in cardiovascular events in patients treated with high-dose ESA, regardless of whether the patient achieved Hb levels (34). CKD can be considered an inflammatory disease. As CKD progresses, the incidence of anemia gradually increases. Roxadustat treatment of anemia is not affected by C-reactive protein (CRP). In two phase III studies in Japan and China, subgroup analysis showed that roxadustat was effective in patients with ESA hyporesponsive anemia. In addition, a 32-person, single-center prospective study found that roxadustat was effective in improving ESA hyporesponsiveness in patients with anemia (35). A case report showed that roxadustat was able to correct renal anemia in patients with anti-EPO antibody-positive patients (36). In addition, the APSN recommends that switching to HIF-PHI should be considered when the cause of ESA hyporesponsiveness due to iron deficiency or other reasons is unknown or difficult to manage. Roxadustat can be an effective drug for the treatment of patients with ESAs hyporesponsiveness. However, most clinical studies have excluded patients with inflammation, and long-term research is still needed in the future.

Disorders of hepcidin and lipoprotein metabolism are considered risk factors for cardiovascular events associated with atherosclerotic disease (37, 38). Studies have shown that high levels of hepcidin reduce iron mobilization and increase iron load in macrophages, thereby reducing endothelial cell survival and leading to endothelial dysfunction. Endothelial apoptosis further leads to plaque erosion and thrombosis (38–40). Furthermore, studies have shown that HIF-1α and HIF-2α are major drivers of endogenous protective mechanisms of cardiac ischemia. PHD inhibition (PHD-i) provides a way to exploit these adaptive responses and enhance the cardioprotective effects of HIF (41). In most cases, increases in HIF expression and PHD-i improve ischemic outcomes (mainly infarct size and cardiac function) (42, 43). HIF-PHI protects the cardiovascular system through multiple physiological effects.

Our meta-analysis showed that roxadustat was associated with AEs of hypertension, hyperkalemia, nausea, diarrhea, and peripheral edema. Patients with CKD may be predisposed to hyperkalemia for a number of reasons, including impaired glomerular filtration rate (GFR), frequent high potassium intake, and use of the renin-angiotensin-aldosterone system (RAS) blocker (44). As CKD progresses, intestinal potassium excretion also increases (45). Despite the increased incidence of hyperkalemia in the roxadustat group, Fishbane S et al. (17) found that the mean post-treatment change in serum potassium in the roxadustat and placebo groups did not differ from baseline, suggesting that roxadustat did not affect serum potassium in the other group. In addition, there may be differences in eGFR between studies, and serum potassium levels may be influenced by dietary differences and RAS blockers. The mechanism by which roxadustat causes hyperkalemia is unknown.

The results of this meta-analysis suggest that roxadustat is associated with an increased incidence of hypertension. However, Fishbane S et al. believed that roxadustat was shown to decrease and increase systolic blood pressure (SBP) and diastolic blood pressure (DBP) by 1 mmHg, respectively, compared to placebo, suggesting that roxadustat did not affect blood pressure (BP) significantly impaired. Shutov E et al. (14) found a higher incidence of elevated SBP in the roxadustat group compared with the placebo group, no significant differences in DBP and 12-lead ECG, and no difference in mean blood pressure (MAP). Significant differences. Animal studies have also shown that roxadustat prevents Ang II-induced hypertension by increasing HIF1α expression to modulate the angiotensin II (Ang II) receptor and eNOS (26). Of course, the possibility that roxadustat can cause hypertension. HIF has a multi-target effects and multiple therapeutic effects, but it cannot avoid AEs. Therefore, AEs should be monitored during treatment. Two-phase IV clinical trials (NCT04059913 and NCT04134026) are underway in China to provide a better clinical reference medication.

Similar to other meta-analyses (12, 13), our results also suggest that roxadustat is effective in improving anemia in NDD-CKD patients. Compared with the two previous meta-analyses (12, 13), we included more studies and performed a detailed analysis of AEs. We found that compared with placebo, although there was no difference between AEs and TEAEs, roxadustat caused some AEs, such as diarrhea and cough. Our study has some limitations. First, all included studies were funded by the pharmaceutical industry and may be biased. Second, we included a small number of studies, one of which had a large sample size, which may have influenced the results of the analysis. In the future, large-sample, multi-center, high-quality clinical trials are crucial to verify the long-term safety of roxadustat and the clinical benefits to patients.

## Conclusion

Roxadustat can increase Hb levels, decrease the hepcidin, SF, TSAT, and increase TIBC, thereby increasing iron utilization and iron storage. In our study, we did not find AEs and severe TEAEs. However, a single AEs analysis found that roxadustat caused symptoms such as cough and diarrhea. Therefore, we believe that roxadustat can be used to treat NDD-CKD anemia under the premise of monitoring mild AEs. Future clinical studies are needed to demonstrate the effect of roxadustat in improving iron metabolism and cardiovascular safety.

## Data availability statement

The original contributions presented in this study are included in the article/Supplementary material, further inquiries can be directed to the corresponding author.

## Author contributions

TC, LX, and CC respectively conducted data extraction, verification, and data analysis. TC performed critical edits to the manuscript and designed the tables and figures. WH performed critical edits to the manuscript, tables and figures, and finalized the content. YT participated in the revision of the article and provided financial support. JH and HD provided technical guidance and revision of the articl. All authors agreed with the manuscript.

## Abbreviations

AEs: adverse events
Ang II: angiotensin II
APSN: Asian Pacific society of nephrology
BP: blood pressure
CKD: chronic kidney disease
CRP: C-reactive protein
DD: dialysis-dependent
DBP: diastolic
ESRD: end-stage renal disease
ESAs: erythropoiesis stimulating agents
EPO: erythropoietin
REPCs: erythropoietin-producing cells
GFR: glomerular filtration rate
Hb: hemoglobin
HIF: hypoxia-inducible factor
HIF-PHIs: hypoxia-inducible factor-prolyl hydroxylase inhibitors
IV: intravenous
IDA: iron deficiency anemia
LDL: low-density lipoprotein
MAP: mean blood pressure
NDD: non-dialysis-dependent
PHD: prolyl hydroxylase domain
RCTs: randomized controlled trials
RR: relative ratio
RAS: renin-angiotensin-aldosterone system
SF: serum ferritin
SBP: systolic pressure
TIBC: total iron-binding capacity
TAST: transferrin saturation
WMD: weighted mean difference.
