# Supplementary material for: Efficacy and safety of roxadustat for the treatment of anemia in non-dialysis chronic kidney disease patients: A systematic review and meta-analysis of randomized double-blind controlled clinical trials
Source: Front Nutr. 2022 Nov 4;9:1029432. doi: 10.3389/fnut.2022.1029432 (PMC9710737; doi:10.3389/fnut.2022.1029432)
Supplement: Supplementary file 1 [file Data_Sheet_1.pdf]

Search terms

PubMed

#1 ("Renal Insufficiency, Chronic"[Mesh]) OR (((((((((Chronic Renal Insufficiencies [Title/Abstract]) OR (Chronic Renal Insufficiency [Title/Abstract])) OR (Chronic Kidney Insufficiency [Title/Abstract])) OR (Chronic Kidney Insufficiencies [Title/Abstract])) OR (Chronic Kidney Diseases[Title/Abstract])) OR (Chronic Kidney Disease[Title/Abstract])) OR (Chronic Renal Diseases[Title/Abstract])) OR (Chronic Renal Disease[Title/Abstract])) OR (renal anemia[Title/Abstract])) Sort by: Publication Date

#2 ("Kidney Failure, Chronic"[Mesh]) OR ((((((End-Stage Kidney Disease [Title/Abstract]) OR (Chronic Kidney Failure [Title/Abstract])) OR (End-Stage Renal Disease [Title/Abstract])) OR (End-Stage Renal Failure [Title/Abstract])) OR (Chronic Renal Failure [Title/Abstract])) OR (ESRD[Title/Abstract])) Sort by: Publication Date

#3 ("FG-4592" [Supplementary Concept]) OR (((Roxadustat [Title/Abstract]) OR (hypoxia-inducible factor prolyl hydroxylase inhibitor[Title/Abstract])) OR (HIF-PHI[Title/Abstract])) Sort by: Publication Date

#4 #1 OR #2

#5 #4 AND #5

Web of science

#1 TS = (Renal Insufficiency, Chronic OR Chronic Renal Insufficiencies OR Chronic Renal

Insufficiency OR Chronic Kidney Insufficiency OR Chronic
WMD Weighted mean difference

Kidney Insufficiencies OR Chronic Kidney Diseases OR

Chronic Kidney Disease OR Chronic Renal Diseases OR

Chronic Renal Disease OR renal anemia)

#2 TS = (Kidney Failure, Chronic OR End-Stage Kidney Disease OR Chronic Kidney Failure OR End-Stage Renal Disease OR End-Stage Renal Failure OR Chronic Renal Failure OR ESRD)

#3 #1 OR #2

#4 TS = (FG-4592 OR roxadustat OR hypoxia-inducible factor prolyl hydroxylase inhibitor OR HIF-PHI)

#5 #3 AND #4
